# Supplementary material for: Resolving the full spectrum of human genome variation using Linked-Reads
Source: Genome Res. 2019 Apr;29(4):635–45. doi: 10.1101/gr.234443.118 (PMC6442396; doi:10.1101/gr.234443.118)
Supplement: Supplemental Material [file supp_gr.234443.118_Supplemental_longranger-2.2.zip › longranger-2.2.tar.bz2!/lr2.2-public/lib/stan/cmdstan/stan/doc/api/html/math_2functions_2log__inv__logit_8hpp_source.html]

Stan: src/stan/math/functions/log\_inv\_logit.hpp Source File


|  |  |
| --- | --- |
|  | Stan  2.5.0  probability, sampling & optimization |


- Main Page
- Related Pages
- Namespaces
- Classes
- Files

- File List
- File Members

All Classes Namespaces Files Functions Variables Typedefs Enumerations Enumerator Friends Macros Pages

- src
- stan
- math
- functions

log\_inv\_logit.hpp

Go to the documentation of this file.

1 #ifndef STAN\_\_MATH\_\_FUNCTIONS\_\_LOG\_INV\_LOGIT\_HPP

2 #define STAN\_\_MATH\_\_FUNCTIONS\_\_LOG\_INV\_LOGIT\_HPP

3

4 #include <boost/math/tools/promotion.hpp>

5

6 namespace stan {

7  namespace math {

8

34  template <typename T>

35  inline typename boost::math::tools::promote\_args<T>::type

36  log\_inv\_logit(const T& u) {

37  using std::exp;

38  if (u < 0.0)

39  return u - log1p(exp(u)); // prevent underflow

40  return -log1p(exp(-u));

41  }

42

43  }

44 }

45

46 #endif

stan::math::log1p

boost::math::tools::promote\_args< T >::type log1p(const T x)

Return the natural logarithm of one plus the specified value.

**Definition:** log1p.hpp:39

stan::math::exp

Eigen::Matrix< T, Rows, Cols > exp(const Eigen::Matrix< T, Rows, Cols > &m)

Return the element-wise exponentiation of the matrix or vector.

**Definition:** exp.hpp:18

stan::math::log\_inv\_logit

boost::math::tools::promote\_args< T >::type log\_inv\_logit(const T &u)

Returns the natural logarithm of the inverse logit of the specified argument.

**Definition:** log\_inv\_logit.hpp:36

stan::agrad::exp

fvar< T > exp(const fvar< T > &x)

**Definition:** exp.hpp:16


---

[ Stan Home Page ]

*© 2011–2014,
Stan Development Team.*
